# Difficulty suppressing visual distraction while dual tasking

**DOI:** 10.3758/s13423-022-02165-2

**Published:** 2022-08-24

**Authors:** John J. McDonald, John M. Gaspar, Hayley E. P. Lagroix, Pierre Jolicœur

**Affiliations:** 1 Department of Psychology, Simon Fraser University, 8888 University Drive, Burnaby, BC V5A 1S6 Canada; 2 Département de Psychologie, Université de Montréal, Montreal, Quebec, Canada

**Keywords:** Dual tasking, Attentional blink, Visual search, Distraction, Distractor Positivity (P_D_)

## Abstract

Human beings must often perform multiple tasks concurrently or in rapid succession. Laboratory research has revealed striking limitations in the ability to dual task by asking participants to identify two target objects that are inserted into a rapid stream of irrelevant items. Under a variety of conditions, identification of the second target (T2) is impaired for a short period of time following presentation of the first target (T1). Several theories have been developed to account for this “attentional blink” (AB), but none makes a specific prediction about how processing of T1 might impact an observer’s ability to ignore a salient distractor that accompanies T2. Using event-related potentials (ERPs) to track target and distractor processing, we show that healthy young adults are capable of suppressing a salient visual-search distractor (D2) while dual tasking (as measured by the P_D_ component, which has been associated with suppression) but struggle to do so shortly after the appearance of T1. In fact, the impairment was more severe for distractor processing than it was for target processing (as measured by the N2pc component). Whereas, the T2-elicited N2pc was merely delayed during the AB, the distractor P_D_ was reduced in magnitude and was found to be statistically absent. We conclude that the inhibitory control processes that are typically engaged to prevent distraction are unavailable while an observer is busy processing a target that appeared earlier.

**Supplementary Information:**

The online version contains supplementary material available at 10.3758/s13423-022-02165-2.

## Introduction

People must often perform multiple tasks concurrently or in rapid succession. Most individuals have an impressive ability to identify objects of interest that appear in rapidly presented streams of visual stimuli (Potter, 1975). Nonetheless, when two objects of interest appear in close temporal succession, identification of the second one is often impaired if the first one is reported correctly. This “attentional blink” (AB) occurs when the time interval between T1 and T2 is 200–500 ms, particularly when at least one irrelevant stimulus intervenes (Duncan et al., 1994; Luck et al., 1996; Martens & Wyble, 2010; Raymond et al., 1992). AB deficits are typically revealed as reductions in response accuracy, but they are also evident as delays in speeded response time (RT; Jolicœur & Dell’Acqua, 1998; Jolicoeur et al., 2001). According to several contemporary theories, attentional processing of T2 is postponed until some process triggered by the appearance of T1 is complete, making the internal representations of T2 vulnerable to decay and interference by trailing stimuli (Jolicœur, 1999; Wyble et al., 2009). By this account, the AB is considered to be a consequence of having insufficient capacity or readiness to attend to the second of two visual targets.

Interestingly, researchers have yet to consider whether processing of T1 might impact an observer’s ability to ignore a salient distractor that accompanies T2. Very few studies have considered how dual-tasking affects our ability to ignore distractors (e.g., Boot et al., 2005) even though distraction is a leading cause of traffic accidents (Regan et al., 2008), poor academic performance (Beland & Murphy, 2016), and workplace inefficiency (Gill et al., 2012). Investigations of inhibitory control processes have risen sharply in the past 2 decades, in part because of the personal and societal costs of having impairment of such processes. Inhibitory control processes contribute to several higher cognitive functions, including the ability to remember relevant information (Engle, 2002; Gaspar et al., 2016; Vogel et al., 2005), and they appear to be particularly susceptible to impairment (Hasher et al., 1999). Aging, anxiety, and attention deficit disorder are all associated with relatively long-term impairments in the ability to suppress visual distractors (Eysenck et al., 2007; Gaspar & McDonald, 2018; Gazzaley et al., 2005; E. Wang et al., 2016). Social jetlag (that is, a misalignment between one’s biological clock and the actual time of day) can disrupt inhibitory control on a circadian (~24 hour) time scale (Smit et al., 2020), but it is unknown whether inhibitory control disruptions occur on the time scale of the AB (that is, within a fraction of a second). The twin aims of the current study were to determine whether healthy young adults are able to suppress a salient visual distractor when required to switch rapidly between two tasks and to determine whether this ability is impaired momentarily after the appearance of a preceding task-relevant target.

**Fig. 1:**
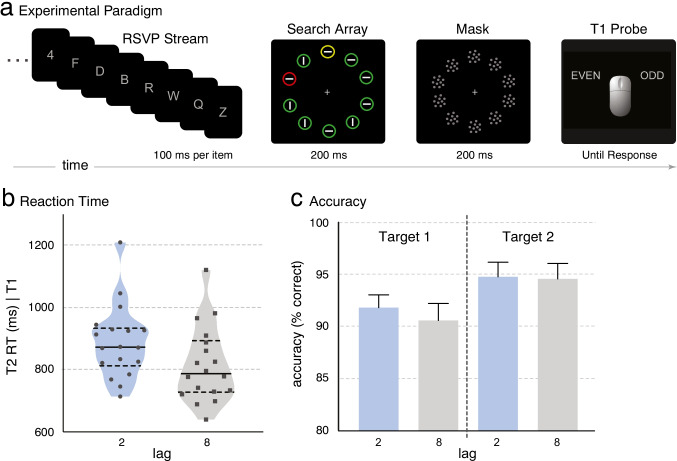
Methods and behavioural manifestation of the AB. **a** The RSVP stream consisted of centrally presented letters (nontargets), a centrally presented digit (T1), a visual search display containing a yellow target (T2) and a red distractor (D2), and a mask display. Following a speeded response to indicate the orientation of a line within the T2 disk, a probe display appeared until participants pressed a button to indicate whether T1 was even or odd. The search display appeared two or eight items after T1 (Lag 2 or Lag 8). T2 and D2 locations were varied to produce lateral-target, midline-distractor displays and midline-target, lateral-distractor displays. **b** Median RTs to T2 (given correct T1 response) for each participant on Lag 2 and Lag 8 trials. Solid lines represent the median across participants, and dashed lines represent the first and third quartiles. **c** Accuracy (% correct) for T1 and T2 tasks at Lag 2 and Lag 8. Error bars reflect the standard errors of the means

Figure 1a depicts the dual task used in the present study. T1 was a centrally presented digit, and T2 was a target of a visual search display that also contained a salient distractor (D2). On each trial, participants first indicated the orientation of the T2 line by means of a speeded button press and then indicated the parity of the T1 when probed to do so. Event-related potentials (ERPs) elicited by the search display were recorded separately on Lag 2 and Lag 8 trials (i.e., when the search display appeared 200 ms or 800 ms after T1) to track target and distractor processing within and beyond the time interval of the AB, respectively. ERPs reflect changes in (primarily cortical) postsynaptic activities that are time-locked to the eliciting event, and thus they enable measurement of neurocognitive processing from stimulus to response (Picton et al., 1995). We isolated lateralized ERP components that have been associated with preattentive salience processing (Ppc; Fortier-Gauthier et al., 2012), attentional selection (N2pc; Eimer, 1996; Luck & Hillyard, 1994) and perceptual-level suppression (P_D_; Gaspar & McDonald, 2014; Gaspelin & Luck, 2018a; Hickey et al., 2009; Sawaki et al., 2012) of D2. If observers are able to suppress a salient distractor after a task switch, D2 should elicit a P_D_ on Lag 8 trials, since observers typically recover from AB deficits within 800 milliseconds. On the hypothesis that T1 processing temporarily disrupts *inhibitory* control processes, we predicted a delay or reduction of the P_D_ on Lag 2 trials but no similar modulation of the pre-attentive Ppc. Finally, we isolated ERP activity associated with target selection and expected to replicate reports of a delay in the target-elicited N2pc at Lag 2 (Lagroix et al., 2015; Pomerleau et al., 2014).

## Materials and methods

The Research Ethics Board at Simon Fraser University (SFU) approved the research protocol used in this study. All experimental procedures were performed in accordance with guidelines and regulations outlined by SFU and the Natural Sciences and Engineering Research Council of Canada.

### Participants

Twenty SFU students participated after giving informed consent. These students were given course credit for their participation as part of a departmental research participation program. All subjects reported normal or corrected-to-normal visual acuity and had normal color vision (tested with Ishihara color plates). Data from two participants were excluded from the analysis due to excessive ocular artifacts (detected on >25% of trials), leaving eighteen subjects in the final sample (eight women; 15 right-handed; mean age 19.6 years ± 2.0 *SD*). The sample size was determined a priori to have sufficient power (1 − β = .80) to detect an effect size that is typical of N2pc and P_D_ studies (Cohen’s *d* = .70) using paired *t* tests. This predetermined sample size had the same power (.80) to detect a difference in N2pc amplitude between Lag 2 and Lag 8, based on the effect size in a previous study with a T2/D2 search task (η_p_^2^ = 0.356; Pomerleau et al., 2014).

### Apparatus

The experiment was conducted in an electrically shielded chamber dimly illuminated by DC-powered LED lighting. Visual stimuli were presented on a 23-inch LCD monitor running at 120 Hz and viewed from a distance of 57 cm. Stimulus presentation was controlled by Presentation (Neurobehavioral Systems, Inc., Albany, CA) from a Windows-based computer. EEG was recorded from a second Windows-based computer connected to a high input impedance EEG amplifier system with active electrodes (Biosemi, The Netherlands). The stimulus-control and acquisition computers were situated outside of the testing chamber.

### Stimulus and procedure

Rapid serial visual presentation (RSVP) streams were comprised of digits and uppercase letters (*x* = 0.30, *y* = 0.36, 8 cd/m^2^) presented centrally on the display. Alphanumeric characters were approximately 1° in height and varied proportionally in width. Visual search arrays were comprised of 10 unfilled rings presented equidistant (9.2°) from a central fixation point. Each ring was 3.4° in diameter with a 0.3° thick outline. Eight of the rings were green nontargets (*x* = 0.29, *y* = 0.64, 8 cd/m^2^), one was a dark-yellow target (*x* = 0.42, *y* = 0.52, 8 cd/m^2^), and one was a red distractor (*x* = 0.64, *y* = 0.32, 7.0 cd/m^2^). The red distractor singleton was the most salient item in the search array due in part to its greater local contrast with surrounding green items (Gaspar & McDonald, 2014). A vertical or horizontal gray line (*x* = 0.30, *y* = 0.36, 8 cd/m^2^; orientation set randomly) was contained within each of the rings. All stimuli were presented on a uniformly black background (0.5 cd/m^2^). Colours were fixed in this study, but a prior study showed that lateralized ERPs elicited by the distractor singleton were determined by relative salience, not colour, at least when chromatic nontargets are used (Gaspar et al., 2016).

Each trial began with an 800–1,200 ms fixation period, during which the central fixation point was visible (Fig. 1a). Participants were instructed to maintain fixation on the central point throughout each trial. The fixation period was followed by a 14-item RSVP stream comprised of 13 letters (nontargets) and a single digit (T1). Letters (A–Z, except I and O) were selected at random with the constraint that the same letter could not appear more than once in a stream. The digit (1–8) was selected at random with the constraint that an equal number of even and odd numbers would be presented within each block. The duration of each alphanumeric stimulus and the stimulus-onset asynchrony (SOA) between successive items in the RSVP stream were fixed at 100 ms. Immediately following the last alphanumeric item in RSVP stream, a visual search display was presented for 200 ms. The yellow and red disks on the search display were T2 and D2, respectively. The spatial positions of T2 and D2 were varied to produce two display configurations: lateral target, midline distractor (50%); midline target, lateral distractor (50%). For each configuration, the lateral singleton (T2 or D2) could appear with equal probability at any one of the eight lateral locations, and the midline singleton could appear with equal probability above or below fixation. The search display was followed immediately by a mask display comprised of 10 circular patches of dots centered on each of the 10 search-display disks. The mask display lasted for 200 ms.

T1 was either the seventh item or the thirteenth item in the RSVP stream. Thus, the search array appeared either eight items after T1 (Lag 8; outside of the period of the AB) or two items after T1 (Lag 2; within the period of the AB). Participants were instructed to first indicate the orientation of the line inside T2 as quickly and as accurately as possible by pressing one of two buttons on a standard computer mouse. Participants were then probed to indicate in unspeeded fashion whether T1 had been an even or an odd number using the same mouse. The next trial began immediately after a response was made to the T1 probe.

Each participant performed in 864 trials. At least 36 practice trials were given to each participant prior to the start of the experiment to learn the tasks and to learn to maintain fixation. Participants were instructed to sit still, relax the jaw, maintain eye fixation on the central stimuli, to blink between trials as necessary, and to stretch during participant-controlled rest periods, which were issued after every block of 36 trials.

### Behaviour

Trials on which the participant responded incorrectly to either T1 or T2 were automatically excluded from the analysis. Trials with anticipatory responses (RT < 100 ms) or excessively slow responses (RT > 1,200 ms) were excluded from analysis (less than 1% of all correct trials). Median RTs to T2 were derived for search displays for each participant (our lab has used medians because they are less affected by outliers compared to means). The means of these median RTs were then computed for both Lag 2 and Lag 8 trials. Differences were statistically assessed using paired t-tests. Next, a repeated-measures analysis of variance (ANOVA) with factors for target–distractor separation (1, 2, 3, and 4 positions) and Lag (2 and 8) was then used to assess search performance. A significant Lag × Separation interaction was expected, on the twin assumptions that (1) distractor suppression would increase RTs for nearby targets relative to more distant targets, and (2) distractor suppression would occur on Lag 8 trials but not on Lag 2 trials. The Omnibus two-factor ANOVA was followed by a polynomial contrast analysis to look for interactions of lag with linear, quadratic, cubic trends across the different levels of separation. Finally, two planned pairwise comparisons were performed to compare RTs at the two most extreme target–distractor separations (1 and 4; see Fig. 4a) for each Lag.

### Electrophysiology

#### Recording and preprocessing

EEG signals were recorded from 34 sintered Ag-AgCl electrodes, using a custom montage that included electrode sites FP1, FP2, AF3, AFZ, AF4, F7, F3, FZ, F4, F8, FC5, FCZ, FC6, T7, C3, CZ, C4, T8, CP5, CPZ, CP6, P7, P3, PZ, P4, P8, PO7, POZ, PO8, O1, OZ, O2, M1, and M2. Horizontal electro-oculograms (EOGs) were recorded using two electrodes positioned 1 cm lateral to the external canthus of each eye, and vertical EOGs were recorded using two electrodes positioned above and below the right eye. All EEG and EOG signals were digitized at 512 Hz, referenced in real time to an active common-mode electrode, and low-pass filtered using a fifth-order sinc filter with a −3 dB cutoff at 104 Hz. Electrode offsets were monitored to ensure the quality of the data. After the data acquisition, EEG data for each channel were high-pass filtered (−3 dB point at 0.05 Hz) and then converted from 24-bit to 12-bit integers. During conversion, the EEG channels were referenced to M2, and the single-ended EOG electrode channels were combined into bipolar HEOG and VEOG channels.

EEG processing and ERP averaging were performed using event-related potential software system (ERPSS; University of California, San Diego). A semiautomated procedure was used to discard epochs of EEG contaminated by blinks, eye movements, or excessive noise using our standard procedure and thresholds. Any trial with an artifact within a 1-s interval commencing 200 ms before onset of the search array was rejected. Artifact-free epochs associated with the two search display configurations of interest were then averaged separately to create ERP waveforms. The resulting ERPs were digitally low-pass filtered (−3 dB point at 32 Hz) and digitally re-referenced to the average of the left and right mastoids. All ERP amplitudes and baselines were computed using a 200 ms prestimulus window. The averaged event-related horizontal EOGs did not exceed 2 μV for any individual participant, indicating their gaze remained within 0.3° of the fixation point for a majority of the trials.

ERPs elicited by lateral-T2 search displays and lateral-D2 search displays were averaged separately for Lag 2 and Lag 8 trials, resulting in four sets of ERPs for each participant. ERPs elicited by search displays containing a target in the left or right visual field were combined in such a way as to produce waveforms recorded contralateral and ipsilateral to T2. Similarly, ERPs elicited by lateral-D2 displays were combined to produce waveforms recorded contralateral and ipsilateral to D2. Contralateral-ipsilateral difference waveforms were then computed by subtracting ipsilateral waveforms from corresponding contralateral waveforms, separately for each pair of lateral electrodes (e.g., PO7 and PO8). Negative voltages were plotted upward so that the N2pc would appear in these difference waveforms as an upward deflection and the P_D_ would appear as a downward deflection.

#### Analysis

All N2pc and P_D_ measurements were taken from contralateral-ipsilateral difference waves recorded at electrodes PO7 and PO8. Except where noted, all statistical tests were performed with two tails. The Ppc elicited by lateral-D2 displays was measured as the mean amplitude within a 120–170 ms window. This window is consistent with previous studies that have typically reported the PPC to occur between 120 and 190 ms (Fortier-Gauthier et al., 2012; Jannati et al., 2013; Pomerleau et al., 2014).

Because some variability in N2pc and P_D_ latencies was expected within and across lags (Gaspar et al., 2016; Gaspar & McDonald, 2014; Lagroix et al., 2015), we opted to measure signed areas within relatively wide time windows for these components (Sawaki et al., 2012). The N2pc to lateral-T2 displays was quantified as the signed negative area within a 225–400-ms time window, whereas the P_D_ to lateral-D2 displays was quantified as the signed positive area within the 200–350-ms time window. The P_D_ measurement window began 25 ms before the N2pc time window because (i) stimulus salience is known to affect the timing of the N2pc and P_D_, and (ii) D2 was more salient than T2 (Gaspar & McDonald, 2014). The presence of each component at each lag was evaluated using a nonparametric permutation approach that aimed to compare the signed area measured from the grand-average waveform to signed areas due entirely to noise (Sawaki et al., 2012). This approach was done because noise contributes to signed area even in the absence of a signal. Briefly, each of the search events of interest (lateral-T2; lateral-D2) were randomly recoded for stimulus lateralization (left, right) to eliminate lateralized ERP signals and thus to enable estimation of noise within the measurement window. This recoding process was done for each subject’s data 500 times to yield 500 different grand-averaged ERPs, which were then used to construct a distribution of signed (positive or negative) area values that would be expected to arise from noise alone if the null hypothesis were true. The observed grand-average N2pc or P_D_ would be considered statistically present if the measured signed area fell beyond the 95th percentile of the corresponding noise distribution. The *p* value for each permutation test was calculated using the following equation (Phipson & Smyth, 2010):

1 $$P=\frac{1+\left( number\ of\ permuted\ values\ge observed\ area\right)\ }{1+ total\ number\ of\ permutations}.$$

Differences in component magnitude across lags were tested using conventional parametric statistics, on the assumption that noise levels would be equal across lags. Signed areas were computed within the N2pc and P_D_ time windows (specified above) for each participant, so that paired *t* tests could be performed. As noted above, each of these signed area measures reflects the sum of the signed areas due to a signal (if present) and random noise. To reduce the impact of noise, we measured the signed area within a prestimulus time window of the same length as the component measurement window (−175–0 ms for target N2pc; −150–0 ms for P_D_), on the premise that fluctuations in the prestimulus baseline would reflect noise alone. We then subtracted the pre-stimulus area from the component area to yield an adjusted signed area that was less biased by noise and computed *t* values based on the adjusted signed areas.

N2pc and P_D_ onset latencies were defined as the latencies within a corresponding measurement window at which each component first reached 25% of its peak amplitude. Onset latencies were measured from Jackknife subaverages rather than from individual-subject waveforms due to the inherent problem of quantifying the latency of a peak that was absent. Statistical tests of onset latencies were performed using a conventional correction for jackknifing (Miller et al., 1998).

### Bayesian statistics

We also reported (inverse) Bayes factors BF_01_ to further test any null results from the classical statistical tests (Keysers et al., 2020). The BF_01_ indicates the strength of the evidence for the null hypothesis, with values larger than one favouring the null over the alternative hypothesis. Generally, BF_01_ values between 3 and 10 are considered as moderate evidence for the null hypothesis. We computed Bayes factors using a default prior of 0.707 in JASP.

## Results

We first set out to determine whether any behavioural manifestation of the AB occurred in our task. AB paradigms typically require two unspeeded responses, but we opted for a speeded visual search task in order to relate our ERP measures to RT effects that have been linked to distraction and suppression (Gaspar & McDonald, 2014). Fortunately, prior studies have shown that with speeded T2 tasks, the AB manifests as an increase in RT at short lags relative to those at long lags (Ghorashi et al., 2007; Jolicœur et al., 2001; Jolicœur & Dell’Acqua, 1998; Lagroix et al., 2015). This is precisely what was observed in the present study (Fig. 1b): Median RTs for correct T2 responses (on trials with correct T1 responses) were 85 ms longer at Lag 2 than at Lag 8 (873 ms vs. 788 ms), *t*(17) = 11.05, *p* < .0001, *d* = 5.21. There was neither a reduction of accuracy at Lag 2 (vs. Lag 8), *t*(17) = 0.50, *p* = .624, *BF*_01_ = 3.68 (Fig. 1c) nor a speed-accuracy trade-off, confirming that the interference in this task was manifest as a delay of one or more processes required to respond to T2. This interference is considered to be a form of AB (Ghorashi et al., 2007; Lagroix et al., 2015), although the paradigm is sometimes called the probe-signal paradigm to differentiate it from the procedures commonly used to investigate the AB (Jolicœur, 1999). The Lag 2 interference occurs because participants are still consolidating T1 into short-term memory and are not yet ready to perform limited-capacity operations on T2 (Ghorashi et al., 2007; Jolicœur, 1999; Jolicœur & Dell’Acqua, 1998; Lagroix et al., 2015).

**Fig. 2:**
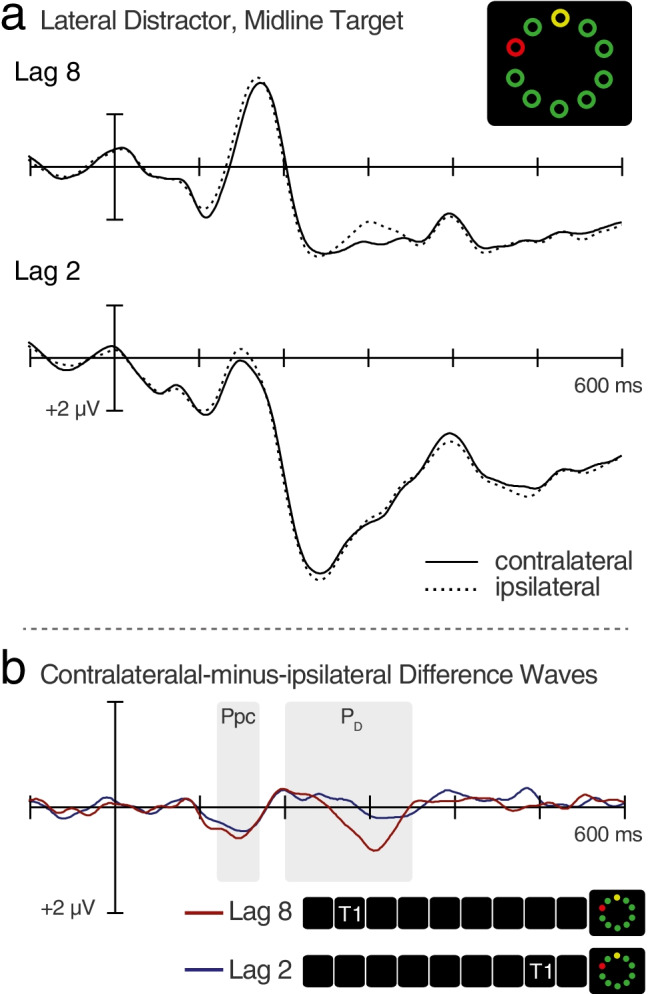
ERPs elicited by lateral-distractor search displays, averaged across the 18 participants. **a** ERPs recorded by occipital electrodes positioned contralateral and ipsilateral to the distractor, separately for Lag 8 trials and for Lag 2 trials. **b** Contralateral-ipsilateral difference waveforms corresponding to the ERPs from (**a**). The distractor-elicited P_D_ appears as a downward deflection in the 200–350 ms time interval

**Fig. 3:**
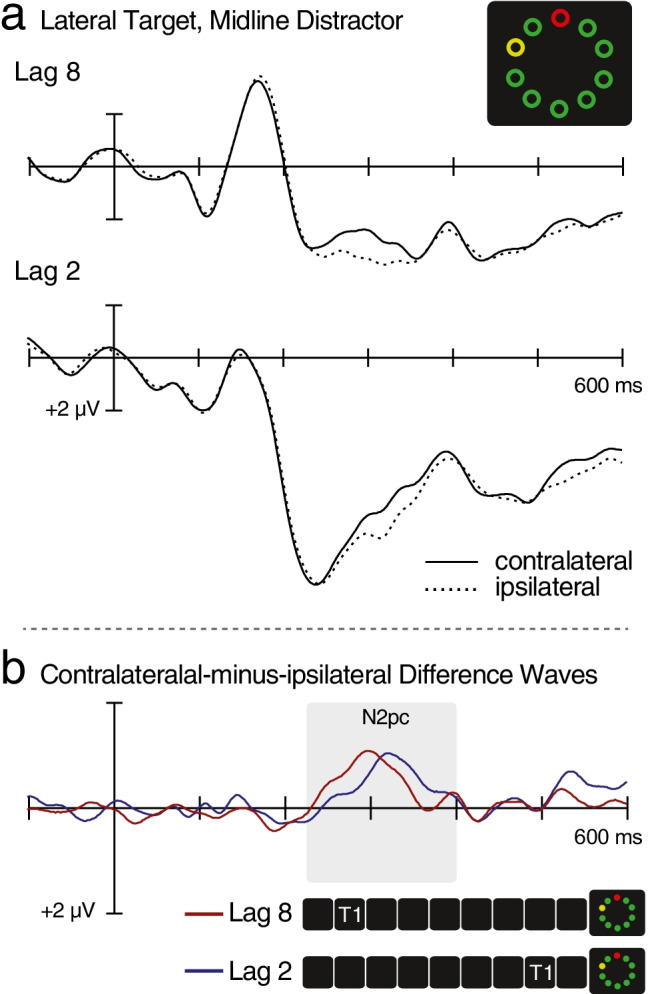
ERPs elicited by lateral-target search displays, averaged across the 18 participants. **a** ERPs recorded by occipital electrodes positioned contralateral and ipsilateral to the target, separately for Lag 8 trials and for Lag 2 trials. **b** Contralateral-ipsilateral difference waveforms corresponding to the ERPs from (**a**). The target-elicited N2pc appears as an upward deflection in the 225–400 ms time interval

After confirming that encoding of T1 slowed processing of T2, we isolated ERPs associated with the selective processing of T2 and D2. On half of the trials, the search array contained a lateral T2 and a midline D2; on the remaining trials, the search array contained a lateral D2 and a midline T2. With such configurations, it is possible to isolate N2pc and P_D_ components elicited by the lateral stimulus, because the midline stimulus cannot elicit lateralized ERP activities associated with attention or suppression (Hickey et al., 2006; Hickey et al., 2009; Woodman & Luck, 1999). At lateral posterior scalp sites, ERPs elicited by each display configuration contained the usual P1 (100–120 ms) and N1 (140–200 ms) components associated with visual processing as well as a later positivity that was larger for Lag 2 trials than for Lag 8 trials (Figs. 2 and 3). This Lag 2 positivity was actually time-locked to T1 rather than to T2, and it appeared to be a P3b that is usually elicited by task-relevant stimuli (see Fig. S1). The N1 appeared to be smaller on Lag 2 trials than on Lag 8 trials, but this was due to overlap with the T1-elicited P3b on Lag 2 trials.

As expected, search displays that contained a lateral D2 were found to elicit the Ppc at Lag 8, *t*(17) = 2.94, *p* = .009, *d* = 1.39, and at Lag 2. *t*(17) = 3.01, *p* = .008, *d* = 1.42 (Fig. 2). Ppc magnitude did not differ across lags, *t*(17) = .64, *p* = .50, *BF*_01_ = 3.43. These findings demonstrate that neither the task switch nor the ongoing identification of T1 interfered with the pre-attentive processing of D2 salience. More importantly, these lateral-D2 search displays were also found to elicit a P_D_ on Lag 8 trials, *p* = .018 (Fig. S2). This finding demonstrates that observers are able to engage the distractor-suppression process underlying the P_D_ even when they must be set for an unrelated (T1) task at the beginning of each trial. Critically, the P_D_ was significantly smaller on Lag 2 trials than on Lag 8 trials, *t*(17) = 2.71, *p* = .015, *d* = 1.28, and its magnitude on Lag 2 trials was no larger than that predicted by the null hypothesis, *p* = .227 (Fig. S2). There was no difference in the magnitudes of the residual horizontal EOG deflections between the two lags, *t* = .94, *p* = .36, and the mean deflections were smaller than 0.5 μV (indicating that eyes were locked onto fixation for the vast majority of trials). Given these findings, we conclude that although healthy young adults can suppress visual distractors after a task switch, they are impaired to suppress distractors for a short time after the appearance of a task-relevant target, presumably because the suppression of D2 requires the engagement of limited-capacity processes that were still fully engaged on T1.

As predicted, search displays containing a lateral T2 were found to elicit an N2pc on Lag 8 trials, *p* = .001 (Figs. 3 and S2). The T2-elicited N2pc was also present on Lag 2 trials, *p* = .001, and although there was no significant difference in N2pc magnitude across lags, *t(*17) = 0.05, *p* = .96, *BF*_01_ = 4.11, the N2pc was found to onset 36 ms later on Lag 2 trials (288 ms) than on Lag 8 trials (252 ms), *t*(17) = 2.5, *p* = .020, *d* = 1.19. N2pc delays of similar magnitude have been interpreted in terms of the difficulty deploying attention to the location of T2 during the period of the AB (Lagroix et al., 2015; Pomerleau et al., 2014). Thus, the present N2pc results provide additional support for the hypothesis that search for T2 is postponed until after T1 processing is complete (Ghorashi et al., 2007; Lagroix et al., 2012; Lagroix et al., 2015; Ptito et al., 2008). The delay in target N2pc accounted for only 42% of the total delay in manual responding, which suggests that multiple sources of interference contribute to the behavioural AB effect and that some of these sources follow the initial deployment of attention. For example, the failure to suppress the distractor may lead to greater response-level conflict at Lag 2 than at Lag 8.

**Fig. 4:**
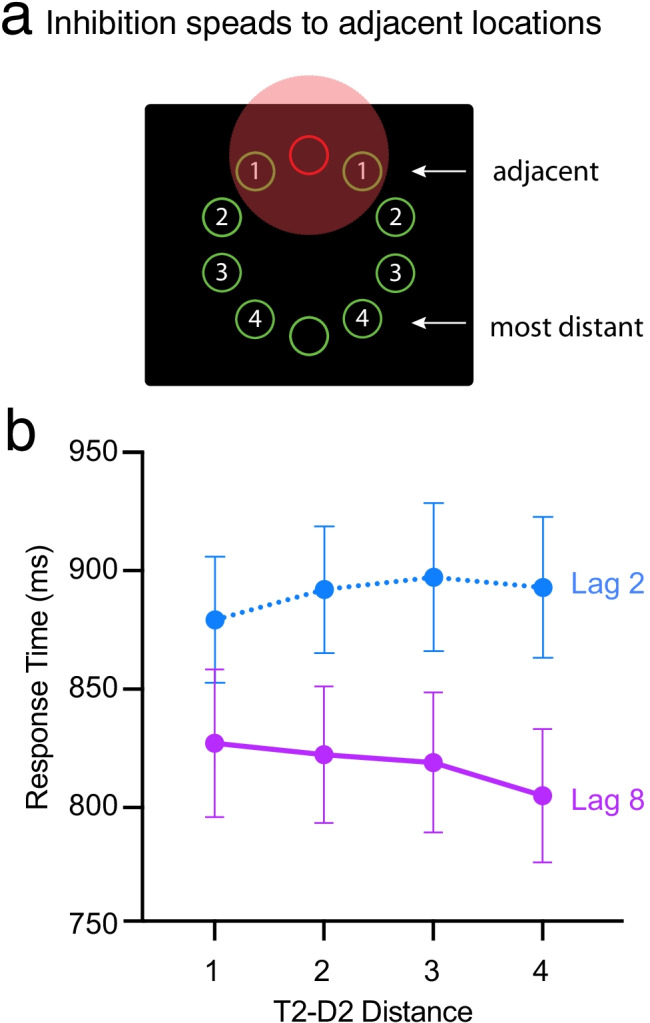
RTs to T2 accompanied by a distractor at different degrees of separation. **a** Illustration of target positions relative to that of a midline distractor. Digits at each lateral position indicate the number of positions separating target and distractor. The red shaded area denotes a zone of inhibition centered upon the distractor. According to the spreading-inhibition hypothesis, RTs will increase as the separation between the two singletons is reduced. **b** Mean RTs for T2 as a function of Lag (Lags 2 and 8) and target-distractor separation (1, 2, 3, and 4 positions). (Color figure online)

Finally, we returned to the RT measures to determine how the impairment in distractor suppression altered performance in this dual task. Under single-task conditions, suppression of a visual-search distractor (as evidenced by the P_D_) aids search for most targets but actually delays search for targets that appear in close spatial proximity to the distractor (Gaspar & McDonald, 2014; Jannati et al., 2013). Presumably this delay happens because some of the visual cortical neurons responding to the two items have large receptive fields, thereby allowing the target to fall within an inhibited region of space when it is near the distractor (Fig. 4a). In colloquial terms, the suppression tied to the location of the distractor spreads to other items, including task-relevant targets, that are within the immediate vicinity of the inhibited location. This spreading inhibition can be measured by comparing RTs to targets that fall next to the distractor with RTs to targets that appear at more distant locations. If participants manage to suppress the distractor, as indexed electrophysiologically by the P_D_, RT should be longer when the target appears next to the distractor than when it appears at a more distant location. In contrast, if participants fail to suppress the distractor during the period of the AB, RTs should not be longer for nearby targets than for more distant targets. Given the P_D_ results obtained here, we predicted to find evidence for spreading inhibition on Lag 8 trials but not on Lag 2 trials.

To test these predictions, we analyzed RTs to T2s as a function of Lag and target–distractor separation. Consistent with the spreading-inhibition hypothesis, mean RTs increased monotonically as the distractor moved closer to the target on Lag 8 trials (Fig. 4b). Critically, however, no such pattern was observed on Lag 2 trials. In fact, at Lag 2, RTs generally *decreased* as the distractor moved closer to the target. In line with this pattern, a repeated-measures ANOVA with factors for Lag (2, 8) and T2–D2 Separation (1, 2, 3, and 4) revealed a significant lag main effect, *F*(1, 17) = 119.4, *p* < .0001, partial η^2^ = .875, and a marginally significant Lag × Separation interaction, *F*(1, 17) = 2.67, *p* = 0.061, partial η^2^ = .136. A polynomial contrast analysis revealed a significant interaction between Lag and the linear trend of separation, *F* (1, 17) = 9.09, *p* = .008, η^2^ = .348, with no other significant trend. This indicates that T2–D2 separation had opposite linear effects on RTs for Lag 2 and Lag 8. Second, planned pairwise comparisons between adjacent-distractor and distant-distractor trials (Separations 1 and 4) revealed that a significant effect of separation on Lag 8 trials, *t* = 2.99, *p* = .008, *d* = 1.41, and a significant-but-reverse effect of separation on Lag 2 trials, *t* = 2.14, *p* = .047, *d* = 1.01. The reverse effect of T2–D2 separation indicates that, during the period of the AB, the salient distractor captured attention on a subset of trials due to an inability to suppress its location, thereby making it easier to find an adjacent target than a more distant target.

## Discussion

The findings of the present study reveal new information about the temporal limits of spatially selective visual processing and, in particular, about the availability of inhibitory control processes while performing two successive tasks. A salient distractor that accompanied T2 was found to elicit the Ppc component at Lag 2 as well as at Lag 8, which indicates that a prioritized salience map of the visual search display was maintained throughout the period of the AB. Healthy young adults retained the ability to search for the target during the time course of the AB but were delayed in orienting attention to the target by 36 milliseconds (as measured by onset latency of the N2pc). This finding confirms that a representation of the T2 search target is maintained while T1 undergoes selective processing at some early stage and is then processed once some critical resources are released from the T1 task (Ghorashi et al., 2007; Lagroix et al., 2015). In contrast, although individuals managed to suppress the distractor at a sufficiently long lag of the dual task, they were less able to do so without sufficient recovery time after T1. Electrophysiologically, the P_D_ was reduced in magnitude (and not statistically significant) at Lag 2. Behaviourally, the typical target-distractor-separation effect on search performance was reversed at Lag 2: Whereas it typically takes longer to identify a target when it appears next to an inhibited distractor (as was the case on Lag 8 trials of the present experiment; Gaspar & McDonald, 2014; Jannati et al., 2013), participants identified the target faster when it was adjacent to the distractor on Lag 2 trials. Together, these findings tell us that participants were less able to suppress the distractor while they continued to process T1 and that search for T2 was biased toward the location of the salient distractor as a result of this failure to suppress.

The major aim of the present study was to determine whether active processing of T1 disrupts *inhibitory* control processes that would otherwise be available to most healthy individuals to help mitigate distraction by a salient but irrelevant visual object. More broadly, we also wanted to know whether those inhibitory control processes could be implemented when observers must switch between two different tasks, because multitasking has become so common in daily life. Prior ERP studies have shown consistently that under single-task conditions, observers can actively suppress salient visual distractors to prevent salience-driven diversion of attention (Drisdelle & Eimer, 2021; Gaspar & McDonald, 2014; Gaspelin & Luck, 2018a), but researchers had not yet considered whether distractor suppression is possible while dual tasking. The findings from the Lag 8 trials of the current study demonstrate that distractor suppression is possible while dual-tasking, as long as there is sufficient time to switch from one task to another. Still, the inability to suppress shortly after processing another task-relevant object underscores the risk of multi-tasking in the presence of visual distractors.

The present conclusions are based on the current understandings of the neurocognitive processes associated with the three lateralized ERP components that were measured in this study. The N2pc is widely believed to be associated with an early stage of selection that helps to isolate an attended item from other items in the display (for a review, see Luck, 2012). At least two findings indicate that the P_D_ is associated with suppression processes that helps to bias attention toward another concurrent item. First, the P_D_ is larger when target discrimination is accomplished quickly than when target discrimination requires more time, indicating that suppression enables observers to process the target more rapidly (Gaspar & McDonald, 2014; Jannati et al., 2013; McDonald et al., 2013). Second, when asked to recall letters overlaid on search items (on randomly intermixed probe trials), participants recall fewer items at the location of a P_D_-eliciting distractor singleton than at a location of an irrelevant nonsingleton shape (Gaspelin & Luck, 2018a). The Ppc has been associated with salience rather than suppression because it occurs whether or not the most salient item in the display (usually a colour singleton) serves as the target or as a distractor (Gaspar & McDonald, 2014; Jannati et al., 2013). When serving as target, the Ppc is followed by an N2pc rather than a P_D_ (as was the case here). Some studies (e.g., Sawaki & Luck, 2010) have attributed the Ppc to suppression rather than salience-driven processing, but few (if any) attempts were made to determine whether Ppc would remain when the eliciting stimulus served as the target rather than as a distractor. Thus, we believe that in the two-singleton task employed here, the Ppc indexes preattentive processes related to salience—the so-called “attend-to-me” signal (Sawaki & Luck, 2010)—rather than subsequent suppression of that signal.

Although the present results show definitive evidence for a distractor-suppression impairment while actively processing the first of two visual targets, it could be argued that the impairment was not specifically related to the AB. This is because the experimental design differed from conventional AB tasks in two ways. First, as noted above, the present task involved a task switch between T1 and T2, whereas the AB persists even in the absence of such a switch (e.g., Chun & Potter, 1995). Second, in the present study, participants made a speeded response to T2 (before making an unspeeded response to T1), whereas conventional AB tasks involve unspeeded responses to both targets. Increases in Lag 2 RTs (vs. Lag 8 RTs) have been linked to the AB in prior studies (e.g., Ghorashi et al., 2007; Lagroix et al., 2015), and along similar lines, we conclude that healthy young adults have difficulty suppressing and ignoring salient visual distractors during the period of the AB. More specifically, we presume that online consolidation of T1 led to the Lag 2 RT delay and the ERP impairments (delay of target N2pc delay, absence of distractor P_D_) in the present study and is a major contributor to AB effects in other, more conventional AB paradigms. Still, future research is needed to determine whether the attentional impairments observed in the present study would persist without a task switch or a speeded T2 response.

The present results have broader implications for our understanding of the inhibitory control processes that help individuals suppress salient visual distractors. Theoretically, the control processes could be completely automatic, completely under volition, or somewhere in between these extremes. Recently, it has been proposed that suppression is due to automatic priming effects because participants appear to be unable to suppress on command (B. Wang & Theeuwes, 2018). Others have suggested that suppression results from *top-down* control processes (Gaspar & McDonald, 2014; Gaspelin & Luck, 2018a), but even these top-down processes were considered to arise from learning and recent experience (i.e., were not considered to be entirely volitional; see Gaspelin & Luck, 2018b). Based on these accounts, one might have predicted distractor suppression to be possible during the period of the AB. The absence of the P_D_ at Lag 2 is directly at odds with such a prediction and with the view that distractor suppression arises from fully automatic processes such as priming. Even though experience undoubtedly contributes to one’s ability to suppress visual distractors, the control processes mediating distractor suppression appear to be dependent on the availability of some critical attentional processes.

## Supplementary information

ESM 1 (DOCX 323 kb)
