# Supplementary material for: Difficulty suppressing visual distraction while dual tasking
Source: Psychon Bull Rev. 2022 Aug 24;30(1):224–34. doi: 10.3758/s13423-022-02165-2 (PMC9971087; doi:10.3758/s13423-022-02165-2)
Supplement: Supplementary file 1 — (DOCX 323 kb) [file 13423_2022_2165_MOESM1_ESM.docx]

Supplementary Materials:

Failure to suppress visual distraction while dual tasking

John J. McDonald^1^*, John M. Gaspar^1^, Hayley E. P. Lagroix^1^, and Pierre Jolicœur^2^

^1^Department of Psychology, Simon Fraser University, Burnaby, BC, Canada, V5A 1S6

^2^Département de Psychologie, Université de Montréal, Montreal, Quebec, Canada

*Corresponding author: John J. McDonald, Department of Psychology, Simon Fraser University, 8888 University Drive, Burnaby, BC, Canada, V5A 1S6. Tel: 778-866-6692. Email**:** jmcd@sfu.ca


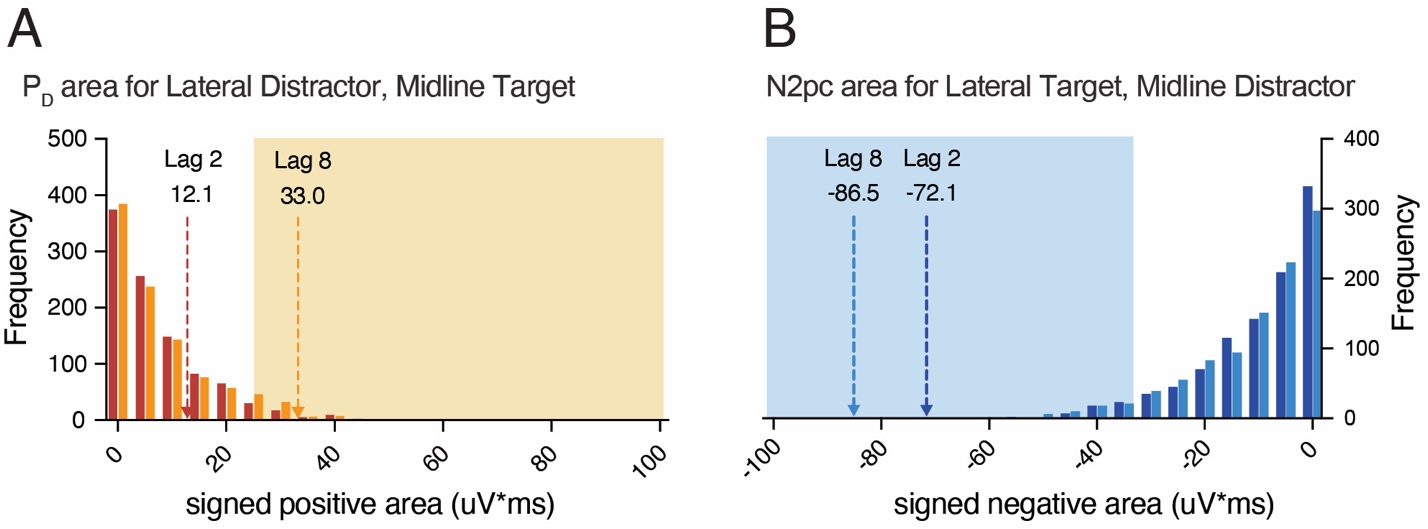


**Fig. S1**. Results of the permutations tests for Lag-2 and Lag-8 trials. Each distribution shows the signed negative or positive area resulting from 500 permutations of the data (i.e., the null distribution). Each shaded area reflects the top five percent of values from the corresponding null distribution. Each arrow reflects the signed area from the actual grand-averaged data. An observed signed area was considered to be statistically different from that expected under the null hypothesis if it fell within the shaded area. (**a**) Signed positive areas within the P_D_ measurement interval on lateral-distractor trials. (**b**) Signed negative areas within the N2pc measurement interval on lateral-target trials.


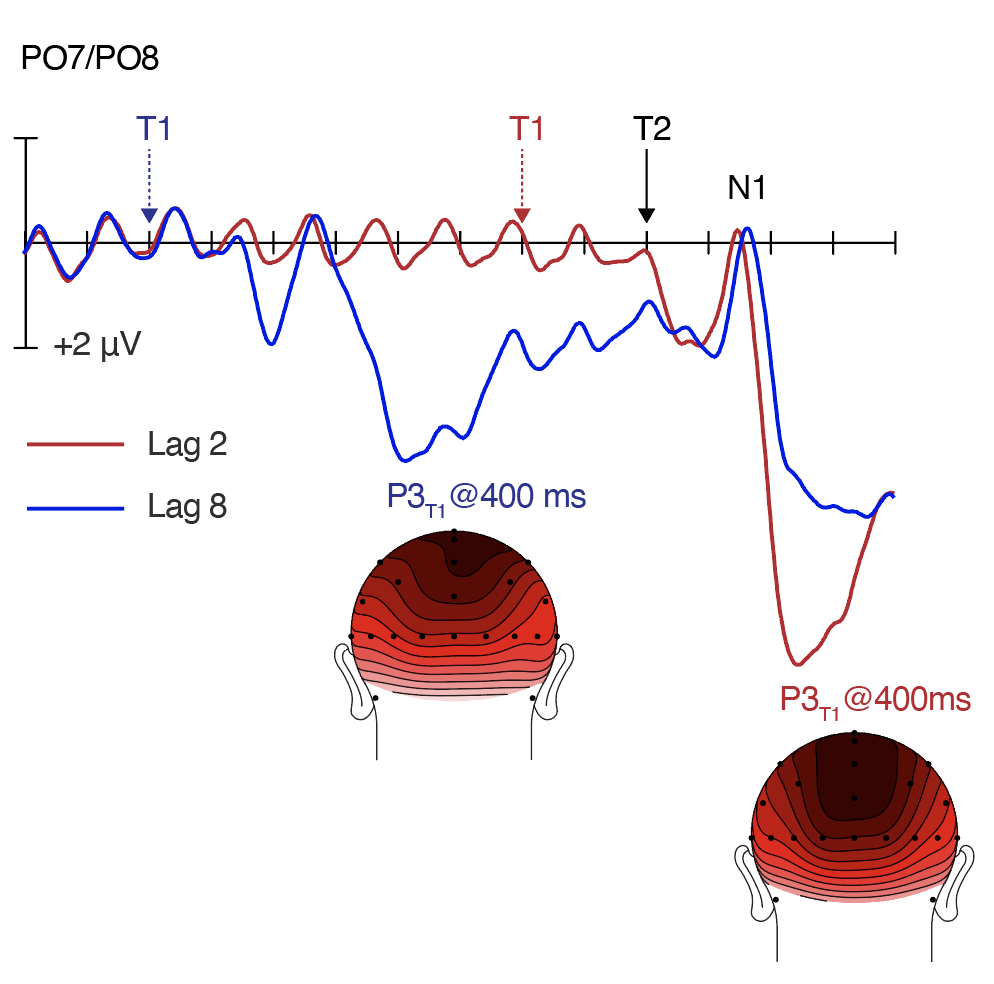


**Fig. S2**. Lateral-occipital ERPs time-locked to T2 but plotted in an interval spanning both T1 and T2. The waveforms were collapsed across the two display configurations (lateral-target; lateral-distractor) and recording hemispheres (contralateral and ipsilateral to lateral singleton) to reveal bilateral activities. A 200-ms interval preceding T1 (rather than T2) was used for baseline correction so that the componentry would be visualized with respect to T1 (and not be shifted differentially due to the presence of the T1-elicited P3b on Lag-8 trials). The T1-elicited P3b peaked roughly 200 ms after the appearance of T2, thereby overlapping with the T2-elicited N1.
